# Proteolytic activation of proapoptotic kinase protein kinase Cδ by tumor necrosis factor α death receptor signaling in dopaminergic neurons during neuroinflammation

**DOI:** 10.1186/1742-2094-9-82

**Published:** 2012-04-27

**Authors:** Richard Gordon, Vellareddy Anantharam, Anumantha G Kanthasamy, Arthi Kanthasamy

**Affiliations:** 1 Parkinson Disorders Research Program, Iowa Center for Advanced Neurotoxicology, Department of Biomedical Sciences, Iowa State University, Ames, IA 50011, USA

**Keywords:** Apoptosis, dopaminergic degeneration, Parkinson’s disease, PKCδ, proteolytic activation, neurotoxicity, neuroinflammation, nuclear translocation, TNFα

## Abstract

**Background:**

The mechanisms of progressive dopaminergic neuronal loss in Parkinson’s disease (PD) remain poorly understood, largely due to the complex etiology and multifactorial nature of disease pathogenesis. Several lines of evidence from human studies and experimental models over the last decade have identified neuroinflammation as a potential pathophysiological mechanism contributing to disease progression. Tumor necrosis factor α (TNF) has recently emerged as the primary neuroinflammatory mediator that can elicit dopaminergic cell death in PD. However, the signaling pathways by which TNF mediates dopaminergic cell death have not been completely elucidated.

**Methods:**

In this study we used a dopaminergic neuronal cell model and recombinant TNF to characterize intracellular signaling pathways activated during TNF-induced dopaminergic neurotoxicity. Etanercept and neutralizing antibodies to tumor necrosis factor receptor 1 (TNFR1) were used to block TNF signaling. We confirmed the results from our mechanistic studies in primary embryonic mesencephalic cultures and in vivo using the stereotaxic lipopolysaccharide (LPS) model of nigral dopaminergic degeneration.

**Results:**

TNF signaling in dopaminergic neuronal cells triggered the activation of protein kinase Cδ (PKCδ), an isoform of the novel PKC family, by caspase-3 and caspase-8 dependent proteolytic cleavage. Both TNFR1 neutralizing antibodies and the soluble TNF receptor Etanercept blocked TNF-induced PKCδ proteolytic activation. Proteolytic activation of PKCδ was accompanied by translocation of the kinase to the nucleus. Notably, inhibition of PKCδ signaling by small interfering (si)RNA or overexpression of a PKCδ cleavage-resistant mutant protected against TNF-induced dopaminergic neuronal cell death. Further, primary dopaminergic neurons obtained from PKCδ knockout (−/−) mice were resistant to TNF toxicity. The proteolytic activation of PKCδ in the mouse substantia nigra in the neuroinflammatory LPS model was also observed.

**Conclusions:**

Collectively, these results identify proteolytic activation of PKCδ proapoptotic signaling as a key downstream effector of dopaminergic cell death induced by TNF. These findings also provide a rationale for therapeutically targeting PKCδ to mitigate progressive dopaminergic degeneration resulting from chronic neuroinflammatory processes.

## Background

Parkinson’s disease (PD) is a debilitating neurodegenerative movement disorder affecting around 2 % of the population over the age of 60 [1,2]. The key pathological hallmark of the disease is a loss of dopaminergic (DA) neurons in the substantia nigra of the midbrain, resulting in a depletion of striatal dopamine that is clinically manifest as a range of motor and non-motor deficits [3]. Recent studies indicate that a sustained neuroinflammatory response is initiated in the substantia nigra pars compacta (SNpc) during early stages of dopaminergic degeneration, and remains evident in postmortem PD brains with increased microgliosis, dystrophic astrocytes and lymphocyte infiltration [4-6]. Compelling evidence over the last decade from animal models, in vitro studies and PD patients has demonstrated that the protracted neuroinflammation that occurs in the SNpc can exacerbate the degeneration of vulnerable dopaminergic neurons. However, the molecular mediators and mechanisms underlying the neuroinflammatory processes remain to be established.

Tumor necrosis factor α (TNF) has recently emerged as an important neuroinflammatory mediator linked to dopaminergic degeneration in PD. Increased levels of TNF are present in the SNpc and the CSF of postmortem PD patients, and genetic polymorphisms in the TNF gene locus have been linked to the development of PD [7-12]. Increased TNF is also well documented in rodent and nonhuman primate models of PD induced by neurotoxic insults and in transgenic models [13,14]. Importantly, ablation of TNF signaling using knockout mice for TNFα or its receptors has been shown to attenuate 1-methyl-4-phenyl-1,2,3,6-tetrahydropyridine (MPTP)-induced dopaminergic degeneration [15,16]. Furthermore, genetic or pharmacological inhibition of soluble TNF signaling with dominant negative mutants is protective against 6-hydroxydopamine and lipopolysaccharide (LPS)-induced dopaminergic degeneration in vivo [17,18]. Conversely, the chronic expression of low levels of TNF in the substantia nigra of rats induced by a viral vector causes progressive dopaminergic degeneration with delayed motor deficits [19]. Together, these independent findings strongly implicate TNF in the progressive loss of dopaminergic neurons in PD. However, the signaling mechanisms by which TNF can mediate dopaminergic degeneration have not been characterized.

Protein kinase Cδ (PKCδ), a member of the novel PKC isoform family, is highly expressed in nigral dopaminergic neurons [20]. Recent studies from our laboratory and others have shown that PKCδ is proteolytically activated by caspase-3 during dopaminergic cell death and that genetic or pharmacological targeting of PKCδ can protect against dopaminergic degeneration in PD models [21-24]. In this paradigm, increased oxidative stress in dopaminergic neurons results in proteolytic activation of PKCδ by caspase-3 downstream of the intrinsic mitochondrial apoptotic pathway. The proteolytic cleavage of PKCδ by caspase-3 persistently activates the kinase as an effector of dopaminergic cell death. However, PKCδ activation by extrinsic neuroinflammatory mediators such as TNF has not been studied and would be relevant to the progression of PD by neuroinflammatory mechanisms. Since TNF also activates caspase-3 by signaling through its death receptors, we determined if PKCδ is proteolytically activated during TNF-induced dopaminergic cell death and in the mouse substantia nigra (SN). Our findings herein identify PKCδ as a key downstream target of TNF death receptor signaling in dopaminergic neurons and demonstrate a novel link between neuroinflammatory mechanisms and progressive dopaminergic degeneration.

## Methods

### Chemicals and reagents

RPMI, neurobasal medium, B27 supplement, fetal bovine serum, l-glutamine, Sytox assay dye, IR-dye-tagged secondary antibodies, penicillin, streptomycin and other cell culture reagents were purchased from Invitrogen (Gaithersburg, MD, USA). Recombinant rat TNF, LPS (*Escherichia coli* 0111:B4) and cytosine arabinoside were purchased from Sigma-Aldrich (St Louis, MO, USA). Recombinant murine TNF and the tumor necrosis factor receptor 1 (TNFR1) neutralizing antibody were from R&D Systems (Minneapolis, MN, USA). Etanercept (Enbrel) was purchased from Amgen, Inc. (Thousand Oaks, CA, USA). Antibodies for rabbit PKCδ and caspase-8 were from Santa Cruz Biotechnology, Inc. (Santa Cruz, CA, USA). Tyrosine hydroxylase (TH) antibody was purchased from Chemicon (Temecula, CA, USA) and microtubule-associated protein 2 (MAP-2) antibody from Cell Signaling Technologies (Beverly, MA, USA). ^32^P-ATP was purchased from Perkin Elmer (Boston, MA, USA) and the AMAXA Nucleofector kit from Lonza (Basel, Switzerland). Caspase assay substrates and inhibitors were purchased from MP Biomedicals (Solon, OH, USA). The DNA fragmentation assay kit was purchased from Roche Applied Science and the Bradford protein assay kit was purchased from Bio-Rad Laboratories (Hercules, CA, USA).

### Culture and treatment paradigm for rat dopaminergic N27 cells

The development and culture conditions of the N27 clonal dopaminergic cell line have been described previously [21,24,25]. Similar culture conditions were used in this study. Briefly, cells were cultured in RPMI 1640 medium containing 10 % heat inactivated fetal bovine serum, 2 mM l-glutamine, penicillin (100 units/ml), and streptomycin (100 μg/ml). Cells were maintained in a humidified atmosphere of 5 % CO_2_ at 37°C. RPMI medium containing 2 % fetal bovine serum was used for the TNF treatment. Cells were washed twice in 2 % RPMI serum and then treated with the indicated doses of recombinant rat TNF.

### Primary mouse mesencephalic neuron cultures

Primary neurons were cultured from ventral mesencephalon tissue of gestational 14-day (E14) mouse embryos, as described previously [21,26] with some modifications. The ventral mesencephalon was dissected under a microscope and collected in ice-cold Dulbecco’s modified Eagle medium F-12 complete medium (DMEM-F12 supplemented with 10 % heat-inactivated fetal bovine serum (FBS), 50 U/mL penicillin, 50 μg/mL streptomycin, 2 mM l-glutamine, 100 μM non-essential amino acids, and 2 mM sodium pyruvate). The tissue was then dissociated using trypsin-ethylenediaminetetra-acetic acid (EDTA) (0.25 %) for 15 minutes at 37°C. Trypsinization was stopped by adding an equal volume of DMEM-F12 complete medium and dissociated tissue was washed in the same medium to remove residual trypsin. The DMEM-F12 medium was aspirated out and the tissue triturated in neurobasal medium containing B-27 antioxidant supplement, 500 μM l-glutamine, 100 U/ml penicillin, and 100 μg/ml streptomycin. After a single cell suspension was obtained, cells were passed through a 70 μm nylon mesh cell strainer to remove tissue debris and aggregates. Cells were counted using a Beckman Coulter ViCell XR automated cell counter and then plated at an equal density (0.8 × 10^6^ cells per well) in 24 well plates containing coverslips precoated with poly-d-lysine (100 μg/ml). Cultures were maintained in neurobasal medium with B-27 antioxidant supplements and cytosine arabinoside (5 μM) was added to inhibit glial proliferation. Cultures were grown in a humidified CO_2_ incubator (5 % CO_2,_ 37°C) and the medium was changed every 2 days. Approximately 4-day-old to 5-day-old cultures were used for treatment. The neuronal cultures were verified to be around 98 % free of glial cells at the time of treatment by using immunocytochemistry for MAP-2, glial fibrillary acidic protein (GFAP) and ionized calcium binding adaptor molecule 1 (iba1) as markers of neurons, astrocytes and microglia, respectively [27]. For TNF treatment, cultures were switched to serum-free neurobasal medium without antioxidant supplements and treated for 48 h. Recombinant murine TNF (30 ng/ml) was added at the beginning of the treatment and re-added again 24 h later. At the end of the treatment, primary cultures were processed for TH immunocytochemistry and neurotransmitter uptake assays.

### DNA fragmentation and Sytox assays

DNA fragmentation was quantified using the Cell Death Detection ELISA Plus assay kit (Roche Applied Science), as described in our previous publications [21,22,24]. This highly sensitive and reliable assay detects and quantifies early changes in apoptosis based on the amounts of histone-associated low molecular weight DNA released into the cytoplasm of cells. Briefly, N27 cells were plated in six-well plates at 0.8 × 10^6^ cells/well and treated the next day with TNF for 16 h. Cells were collected using a cell scraper and centrifuged at 400 × g for 5 minutes. The cells were gently lysed using the lysis buffer provided with the kit. The lysate then was spun down again at 200 *g* for 10 minutes to pellet the nuclear fraction, and the supernatant was collected and used to measure DNA fragmentation according to the manufacturer's instructions for the ELISA protocol. The absorbance at 405 nm was measured against an 2,2'-azino-bis(3-ethylbenzothiazoline-6-sulfonic acid (ABTS) solution as a blank (reference wavelength approximately 490 nm) using a Synergy-2 Multi-Mode Microplate Reader (BioTek Instruments, Inc). The absorbance values were normalized to the amount of protein present in the lysates and the data expressed as percent control. The Sytox cytotoxicity assay was performed using the Sytox green dye (Molecular Probes). The assay is based on the principle that live cells with intact plasma membranes can exclude the Sytox dye, which selectively enters cells with a compromised membrane and emits bright green fluorescence on binding to DNA [28]. N27 cells were grown in 24 well plates and 1 μM of the Sytox dye was added at the time of treatment. Cells were treated for 16 h with TNF and pretreated with etanercept. The increase in green fluorescence, as a result of TNF cytotoxicity, was measured using a fluorescence microplate reader (Spectramax Gemini, Molecular Devices) at an excitation of 485 nm and 538 nm emission. Phase contrast and fluorescence images of matching fields were captured on the same cells to visualize the toxicity in N27 cells.

### Caspase-3 enzymatic activity assays

Enzymatic assays for caspase-3 activity were performed as described previously [22,24] using acetyl-DEVD-amino-4-trifluoromethylcoumarin (Ac-DEVD-AFC, 25 μm) as the fluorometric substrate for the reaction. N27 cells were treated with TNF (30 ng/ml) for 6 h or pretreated with etanercept (5 μg/ml) for 30 minutes. Following treatment, 100 μl of cell extract was incubated with 5 μl of the fluorescence substrate and then incubated at 37°C for 1 h while being protected from light. The fluorescence signal generated upon cleavage of the AFC peptide substrate by caspase-3 was measured at 510 nm with an excitation of 400 nm using a Synergy-2 Multi-Mode microplate reader. Protein concentrations were determined using the Bradford assay. Raw values were normalized using protein concentrations and expressed as percent control.

### Confocal immunofluorescence microscopy for PKCδ translocation

N27 dopaminergic cells were plated on poly-d-lysine (100 μg/ml) coated coverslips in 24 well plates at 0.2 × 10^6^ cells per well. The next day, the cells were treated with 30 ng/ml of TNF for 6 h in 2 % serum RPMI medium. Cells were fixed in 4 % paraformaldehyde for 20 minutes and washed five times in PBS. The cells were blocked and permeabilized with blocking buffer (5 % goat serum, 0.2 % Triton X, and 0.05 % Tween-20 in PBS) for 1 h and then incubated overnight at 4°C with a rabbit polyclonal PKCδ antibody (1:1,000) that recognizes a C-terminal epitope present in both the native and the proteolytically cleaved protein. The cells were then washed five times in PBS and then incubated with secondary antibody (1:2,000, Alexa 488 goat anti-rabbit) for 1 h at room temperature. Negative controls for non-specific staining that contained secondary antibody alone were used on parallel wells to ensure specificity of the fluorescent signals obtained. The cells were then washed five times in PBS and the nucleus was labeled using the TOPRO 3 dye. The cells were then washed three more times in PBS and coverslips were mounted using the ProLong Gold antifade reagent (Molecular Probes). Confocal images were acquired with a Nikon EZ-C1 confocal system using the 488 nm and 633 nm lasers to visualize PKCδ and TOPRO 3, respectively. Fluorescence spatial intensity plots along the XY plane for PKCδ localization (green channel) and the nucleus (blue channel) were generated using the Nikon EZC1 software to show nuclear translocation.

### Fluorescent Western blotting

Lysates from N27 cells and brain tissue were prepared using a modified radio immunoprecipitationassay (RIPA) buffer and normalized for equal amounts of protein using the BCA protein assay (Pierce Biotechnology). Gel loading dye was added to the lysates and stored in aliquots at −80°C. Equal amounts of protein (30 to 60 μg) were loaded for each sample and separated on either 12 % or 15 % SDS-PAGE gels, depending on the molecular weight of the target protein. After separation, the proteins were transferred to nitrocellulose membranes and non-specific binding sites were blocked by incubating the membranes in fluorescent Western blocking buffer (Rockland Immunochemicals) for 1 h, and then they were probed with primary antibodies overnight at 4°C. Primary antibodies used were rabbit polyclonal PKCδ (1:500), mouse monoclonal TH (1:2,000), rabbit polyclonal caspase-8 (1:200). β-Actin (1:5,000) was used as the loading control. After incubation, membranes were washed three times with PBS containing 0.05 % Tween and IR-dye tagged secondary antibodies (1:5,000; Molecular Probes) were added. Membranes were visualized on the Odyssey infra-red imaging system.

### PKCδ immunoprecipitation (IP)-kinase assays

The PKCδ enzymatic kinase activity assay was performed as described previously [21,22,29]. Briefly, N27 cells or substantia nigra tissues were washed in ice-cold PBS and then resuspended in a mild RIPA lysis buffer containing protease and phosphatase inhibitor cocktail (Pierce Biotechnology). The lysates were placed on ice for 20 minutes, sonicated gently and centrifuged at 13,000 rpm for 45 minutes. The supernatant was collected and protein concentration was determined using the Bradford assay [30]. All samples were made up to a concentration of 2 μg/ml, and 500 μg of total protein in a 250 μl volume was immunoprecipitated overnight at 4°C using 5 μg of the PKCδ antibody. The next day, the protein A-agarose beads (Sigma-Aldrich) were added and the samples were incubated for 1 h at room temperature. The protein A-bound antibody complexes were then washed three times in 2 × kinase assay buffer (40 mM Tris, pH 7.4, 20 mM MgCl_2_, 20 μM ATP, and 2.5 mM CaCl_2_), and then resuspended in 40 μl of the same buffer. The kinase reaction was started by adding 40 μl of the reaction buffer containing 0.4 mg of histone H1, 50 μg/ml phosphatidylserine, 4 μM dioleoylglycerol, and 10 μCi of [γ-^32^P] ATP at 3,000 Ci/mM to the immunoprecipitated samples. The samples were incubated for 10 minutes at 30°C. The kinase reaction was stopped by adding 2 × SDS loading buffer and boiling the samples for 5 minutes. The proteins were separated on a 12 % SDS-PAGE gel and the phosphorylated histone H1 bands were scanned using a Fujifilm FLA 5000 imager. Image analysis and band quantification were performed using the Fujifilm Multigauge software package (Fujifilm USA, Stamford, CT).

### Transfection with siRNA and cleavage-resistant PKCδ^D327A^-CRM mutant

Design and synthesis of PKCδ siRNA are described in our previous publications [21,31]. N27 cells were transfected with 40 to 50 nM of either PKCδ or scramble siRNA duplexes using the AMAXA Nucleofector kit, according to the manufacturer’s instructions. Transfected cells were counted using a Vi-Cell XR automated cell counter and seeded at equal density (0.5 × 10^6^ cells/well) into six-well plates. The cells were treated 24 h after transfection to allow for optimal knockdown of gene expression. At the end of the treatment, cells were collected and used for the DNA fragmentation assay as described above. For studies with the PKCδ cleavage-resistant mutant (PKCδ^D327A^-CRM), stable N27 cell lines overexpressing either the mutant PKCδ^D327A^ protein or the β-galactosidase (Lac Z) protein as the vector control were prepared as described in our publications [32]. Both cell lines were cultured in the presence of blasticidin for three passages before treatment. Expression of the PKCδ CRM-mutant and the Lac Z vector control at the time of treatment was confirmed by staining for the V5-epitope. Cells were plated at equal density (0.7 × 10^6^ cells per well) in six-well plates and allowed to grow overnight. The next day, cells were treated with 30 ng/ml of TNF for 16 h and processed for the DNA fragmentation assay.

### Dopamine uptake assays

Uptake assays for tritiated dopamine on primary EVM cultures were performed according to previously published protocols [17,33,34]. After TNF treatment, the primary neuron cultures from PKCδ wild type (+/+) and knockout (−/−) mice were washed three times with 0.5 ml of warm Krebs-Ringer buffer (16 mM sodium phosphate, 120 mM NaCl, 4.7 mM KCl, 1.8 mM CaCl_2_, 1.2 mM MgSO_4_, 1.3 mM EDTA, and 5.6 mM glucose; pH 7.4). The cultures were then incubated with 5 μM ^3^ H]-dopamine (30 Ci/mmol) in Krebs-Ringer buffer at 37°C for 20 minutes. The cells were then washed three times with ice-cold Krebs-Ringer buffer and collected in 1 ml of 1 N NaOH. Scintillation fluid was added to a total volume of 5 ml and radioactivity counts were measured using a Tri-Carb Liquid Scintillation counter (Packard). In parallel wells, the nonspecific uptake of dopamine was determined by incubation with 10 μM mazindol. The nonspecific uptake values were subtracted to obtain the data for high affinity neurotransmitter uptake. Data from dopamine uptake studies were expressed as percent control.

### TH-positive cell counts and immunofluorescence

After TNF treatment, primary neuron cultures from PKCδ wild type (+/+) and PKCδ knockout (−/−) mice were fixed in 4 % paraformaldehyde and permeabilized with PBS containing 2 % bovine serum albumin (BSA), 0.2 % Triton X-100 and 0.05 % Tween-20. Blocking buffer (PBS with 2 % BSA) was added for 1 h at room temperature and primary antibodies for TH (1:1,000) and MAP-2 (1:1,000) were diluted in blocking buffer and incubated overnight at 4°C. The next day, cells were washed in PBS four times and incubated with appropriate Alexa-dye conjugated secondary antibodies for 1 h at room temperature. After several washes, the samples were counterstained with Hoechst to label the nucleus, and coverslips were mounted using the Prolong Antifade (Molecular Probes) mounting medium. Images were acquired using a Nikon inverted fluorescence microscope (model TE-2000U) equipped with a SPOT digital camera system (Diagnostic Instruments, Sterling Heights, MI, USA). Image analysis was performed using the Metamorph software package (Universal Imaging Systems, PA, USA).

### Stereotaxic infusion of LPS into the mouse substantia nigra

A single dose LPS injection model that was previously described [35] was used to induce delayed, progressive loss of dopaminergic neurons in the substantia nigra. C57/BL/6 mice (8 weeks old, n = 6 per group) were anesthetized with a mixture of ketamine-xylazine (100 mg/kg, 10 mg/kg) and carefully immobilized on a stereotaxic apparatus (Benchmark Angle One, Leica Microsystems). The skin above the skull was prepped with alcohol and an incision was made to expose the skull. A single burr hole was carefully drilled at the injection site for the right SN and ophthalmic gel was used to protect the eyes. The stereotaxic coordinates for the injection site were anteroposterior (AP) -3.3 mm, mediolateral (ML) -1.2 mm, dorsoventral (DV) -4.6 mm from bregma [36]. A stainless steel cannula attached to a 5 μl Hamilton syringe was carefully inserted into the hole drilled at the injection site and a single dose of 5 μg LPS in a 1 μl volume was injected at the rate of 0.5 μl per minute. The needle was left in place for another 5 minutes to prevent retrograde flow of liquid along the needle track. Control mice were injected in an identical manner with equal volumes of saline. After surgery, the skin was sutured and carefully held in place using sterile, non-pyrogenic stainless steel clips (Autoclip Wound Closing System, Braintree Scientific, Inc). Mice were allowed to recover on a heating pad (Braintree Scientific, Inc) and were carefully monitored through recovery from anesthesia. All animal procedures were approved by the Iowa State University Institutional Animal Care and Use Committee (IACUC).

### Data analysis

Data analysis was performed using the Prism 4.0 software package (GraphPad Software, San Diego, CA, USA). The data were first analyzed using one-way analysis of variance (ANOVA) and then Bonferroni’s post-test was performed to compare all treatment groups. Differences of *P* <0.05 were considered statistically significant. The Student’s *t* test was used when differences between two groups were being compared.

## Results

### TNF is neurotoxic to dopaminergic N27 cells and activates caspase-8 and caspase-3

**Figure 1:**
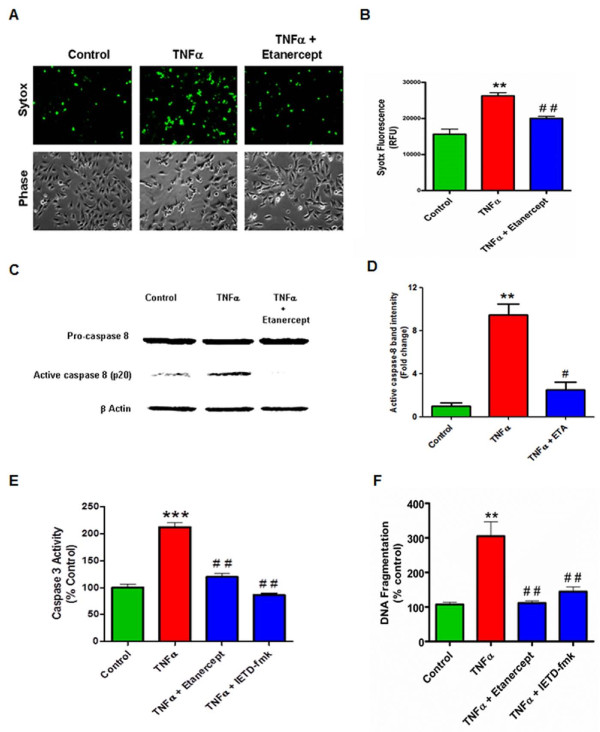
**Tumor necrosis factor (TNF)-induced neurotoxicity and caspase activation in N27 dopaminergic neuronal cells. (A)** Visualization of TNF toxicity by Sytox fluorescence cytotoxicity assay. N27 dopaminergic cells treated with TNF (30 ng/ml), or pretreated with etanercept (5 μg/ml) for 30 minutes and were processed for Sytox green imaging at 16 h. Increased green fluorescence is evident with TNF treatment, indicating significant neurotoxicity which was also evident in the phase contrast images. **(B)** Sytox fluorescence was quantified by measuring the fluorescence intensity as relative fluorescence units (RFU) using a plate reader. **(C)** Activation of caspase-8 by TNF treatment. Lysates from N27 cells treated with TNF (30 ng/ml) for 3 h were probed using a rabbit polyclonal antibody to caspase-8 that detects the procaspase and the active cleaved form. TNF induced a significant increase in the active caspase-8 (p20) fragment, which was blocked by pretreatment with etanercept (5 μg/ml). **(D)** Band intensities for cleaved caspase-8 from three blots were quantified using densitometric analysis and normalized to β-actin. **(E)** TNF induced activation of caspase-3. Enzymatic assays for caspase-3 activity in lysates from N27 cells treated with TNF (30 ng/ml) for 6 h or pretreated with either etanercept (5 μg/ml) or the caspase-8 inhibitor IETD-fmk (25 μM) for 30 minutes. Raw values were normalized using protein concentrations and expressed as percent control. TNF treatment induced significant activation of caspase-3, which was blocked by etanercept and the caspase-8 inhibitor IETD-fmk. **(F)** TNF-induced DNA fragmentation. N27 cells were treated with TNF (30 ng/ml) for 16 h or pretreated for 30 minutes with either etanercept (5 μg/ml) or the caspase-8 peptide inhibitor IETD-fmk (25 μM), and processed for the DNA fragmentation assay. Raw values were normalized using protein concentration and expressed as percent control. TNF treatment significantly increased DNA fragmentation, which was attenuated by etanercept or caspase-8 inhibition. Data represent the group mean ± SEM; n = 6 to 8 per group and experiments were repeated three times. ** (*P* <0.01) indicates significant differences compared to control cells; ## (*P* <0.01) and # (*P* <0.05) indicates significant differences compared to TNF-treated cells.

To determine the downstream mechanisms underlying TNF stimulation in dopaminergic neuronal cells, we used the N27 dopaminergic neuronal cell model, which has been widely used by several laboratories to study dopaminergic degeneration related to PD [21,24,25]. First we examined the neurotoxic effect of TNF on N27 dopaminergic neuronal cells. The Sytox assay and the sensitive DNA fragmentation ELISA were used to quantify TNF cytotoxicity. TNF (30 ng/ml) induced significant increases in Sytox fluorescence and cell death morphology, which could be blocked using the TNF neutralizing drug etanercept (Figure 1A,B). Since TNF cytotoxicity can proceed through activation of caspases in various cell types, we examined caspase activation downstream of TNF signaling. High levels of the pro-caspase-8 protein were detected in N27 cells, and TNF treatment induced activation of caspase-8 at 3 h, with increased accumulation of the active p20 fragment (Figure 1C,D). Again, the activation of caspase-8 was attenuated by pretreatment with the TNF inhibitor etanercept. Since caspase-3 is an important effector caspase downstream of TNF signaling, and is known to be activated in PD models and in the human PD brain [37,38], we studied caspase-3 activation in TNF-treated N27 cells. We found that TNF treatment for 6 h caused activation of caspase-3, which could be blocked by pretreatment with etanercept and the caspase-8 inhibitor IETD-fmk (Figure 1E). These results indicate that caspase-3 is activated in a caspase-8 dependent manner downstream of TNF death receptor signaling in dopaminergic neuronal cells. Next we used the sensitive DNA fragmentation ELISA assay to quantify TNF cell-death signaling. Exposure to TNF (30 ng/ml) for 16 h resulted in a threefold increase in DNA fragmentation compared to untreated controls (Figure 1F). Pretreatment with the TNF-neutralizing drug etanercept or a caspase-8 inhibitor (IETD-fmk) almost completely blocked TNF-induced DNA fragmentation, indicating that canonical TNF death receptor signaling through activation of caspase-8 is involved in the dopaminergic neurotoxicity caused by sustained exposure to TNF. In preliminary studies, we found that doses as low as 10 ng/ml TNF could induce detectable toxicity in N27 dopaminergic cells. We chose a 30 ng/ml dose of TNF based on previous in vitro studies [26] and because this concentration induced a consistent neurotoxic response in vitro. The dose range of TNF used in our mechanistic studies is high compared to the levels of circulating TNF in the serum and CSF of PD patients [39-41] due to the fact that high doses are necessary to elicit a detectable response in cell culture models within a shorter timeframe. Also, the circulating TNF has a short half-life and therefore, it does not reflect the elevated levels of tissue TNF in the substantia nigra. The TNF concentration at 10 to 60 ng/ml used in our study is consistent with doses used in cell culture experiments by other researchers [17,26].

### TNF induces proteolytic activation of protein kinase Cδ in dopaminergic neuronal cells

**Figure 2:**
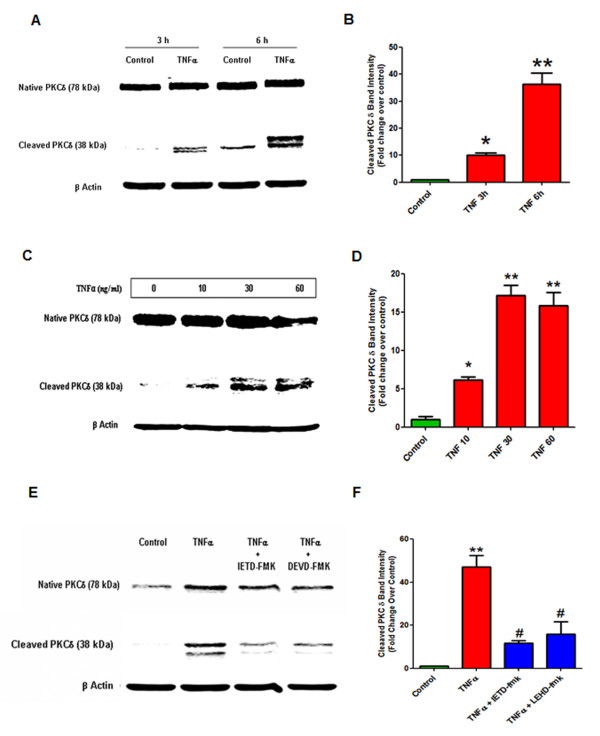
**Tumor necrosis factor (TNF) induced proteolytic cleavage of protein kinase Cδ (PKCδ) in dopaminergic N27 cells. (A)** Time-dependent PKCδ proteolytic activation. N27 dopaminergic cells were treated with recombinant TNF (30 ng/ml) for 3 h and 6 h and lysates were probed by Western blotting with an antibody to the C-terminus that detects both the native protein (78 kDa) and the proteolytically cleaved catalytic fragment (38 kDa). TNF treatment caused a time-dependent increase in PKCδ cleavage, which peaked at 6 h. **(B)** Band intensities for cleaved PKCδ from three blots quantified using densitometric analysis and normalized to β-actin. **(C)** Dose-dependent proteolytic activation of PKCδ by TNF treatment. N27 cells were treated with increasing doses of TNF (0 to 60 ng/ml) for 6 h and lysates were probed for PKCδ by Western blotting. TNF induced a dose dependent increase in PKCδ cleavage, starting at 10 ng/ml. **(D)** Band intensities for cleaved PKCδ from three blots were quantified using densitometric analysis and normalized to β-actin. **(E)** Caspase-3 and caspase-8 dependent proteolytic cleavage of PKCδ. N27 cells were treated with 30 ng/ml of TNF alone for 6 h or in the presence of peptide inhibitors (25 μM) of caspase-8 (Ac-IETD-fmk) and caspase-3 (Ac-DEVD-fmk). Lysates were probed for PKCδ proteolytic cleavage by Western blotting. Proteolytic activation of PKCδ by TNF treatment was reduced by inhibition of caspase-3 and caspase-8. **(F)** Band intensities for cleaved PKCδ from three blots were quantified using densitometric analysis and normalized to β-actin. Data is expressed as a fold change over control. * (*P* <0.05) and ** indicate significant differences compared to control cells; # (*P* <0.05) indicates significant differences compared to TNF-treated cells.

We recently showed that PKCδ, a proapoptotic kinase belonging to the novel PKC isoform family, is highly expressed in dopaminergic neurons [21,42]. Further, we demonstrated that PKCδ is activated by caspase-3 mediated proteolytic cleavage at its hinge region following mitochondrial oxidative stress, resulting in persistent activation of the kinase and amplification of apoptotic signals in dopaminergic neurons [21,22,24]. Since PKCδ activation by the extrinsic apoptotic pathway in dopaminergic cells or its role in neuroinflammation-induced dopaminergic neurotoxicity has not been examined, we tested if PKCδ can be activated by caspase-3 during TNF-induced death of dopaminergic neuronal cells and function as a downstream apoptotic effector. Interestingly, TNF treatment caused a time and dose dependent proteolytic activation of PKCδ that was detectable 3 h after treatment and increased at 6 h (Figure 2A,B). Dose response studies at the 6 h time point showed that doses as low as 10 ng/ml of TNF could induce detectable proteolytic activation of PKCδ, which increased substantially with 30 ng/ml TNF. Higher doses of TNF (60 ng/ml) did not significantly increase PKCδ cleavage further (Figure 2C,D). Since caspase-8 and caspase-3 were activated by TNF in these cells, we tested if PKCδ proteolytic activation was mediated by this signaling pathway using the specific caspase inhibitors DEVD-fmk and IETD-fmk. Pretreatment with the caspase-8 and caspase-3 inhibitors suppressed TNF-induced proteolytic activation of protein kinase Cδ (Figure 2E,F). Taken together, these results suggest that the proapoptotic kinase PKCδ could be activated by caspase-8 and caspase-3 dependent proteolytic cleavage during TNF toxicity in dopaminergic neuronal cells. The proteolytically cleaved PKCδ fragment appeared as a doublet band in Western blots in our experiments. Several alternate splice forms of PKCδ have been reported, including a caspase-3 cleavage-resistant form and a truncated form lacking the regulatory domain [43,44]. Also, the C-terminal catalytic domain of PKCδ contains multiple phosphorylation sites. Because the antibody used in these studies recognizes the C-terminal catalytic domain, it is possible that the doublet band of the cleaved product represents a post-translational modification of the cleavage product or one of the truncated PKCδ alternate splice forms.

### Proteolytic activation of PKCδ by TNF signaling activates the kinase

**Figure 3:**
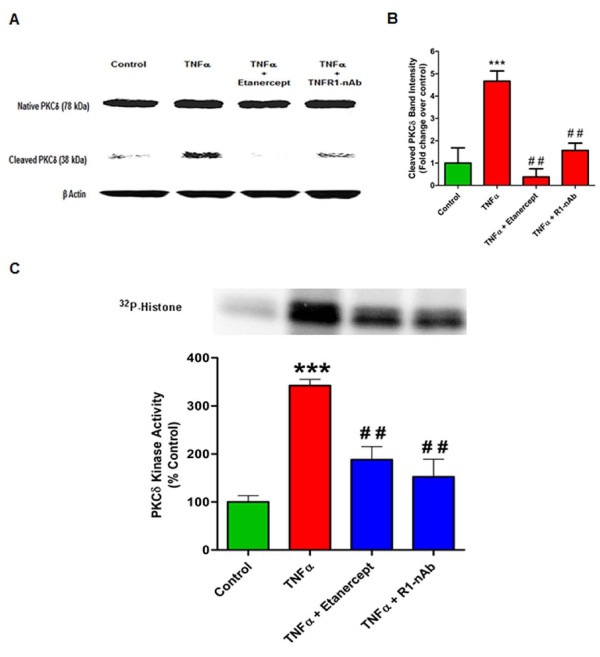
**Proteolytic cleavage and activation of protein kinase Cδ (PKCδ) downstream of tumor necrosis factor receptor 1 (TNFR1) in N27 dopaminergic cells. (A)** TNFR1-dependent proteolytic activation of PKCδ. N27 cells were treated with tumor necrosis factor (TNF) (30 ng/ml) for 6 h or pretreated with either a TNFR1 neutralizing antibody (TNFR1-nAb, 20 μg/ml) or etanercept (5 μg/ml) for 30 minutes and lysates were probed for PKCδ proteolytic cleavage by Western blotting. TNF induced strong proteolytic cleavage of PKCδ that could be blocked by neutralizing TNFR1 receptor signaling. **(B)** Band intensities for cleaved PKCδ were quantified using densitometric analysis and normalized to β-actin levels. **(C)** Immunoprecipitation (IP)-kinase assay for PKCδ activation. IP-kinase assays were performed on the same cell lysates used for Western blots. PKCδ was immunoprecipitated from 500 μg of total protein from each sample and used for the in vitro kinase activity assay with a histone substrate and radiolabeled [γ-^32^P] ATP. PKCδ activity was quantified using densitometric analysis of the ^32^P-histone band intensity and expressed as percent control. The proteolytic cleavage of PKCδ induced by TNF treatment was accompanied by a concomitant increase in kinase activity and was dependent on signaling through the TNFR1 receptor. A representative kinase assay gel is shown. Data represent the group mean ± SEM of densitometric values obtained from three independent experiments. *** (*P* <0.001) denotes significant differences compared to control cells and ## (*P* <0.01) denotes significant differences compared to TNF-treated cells.

Unlike other proteins for which proteolytic cleavage can result in loss of function or inactivation, the proteolytic cleavage of PKCδ by caspase-3 during oxidative stress-induced dopaminergic cell death has been shown to persistently activate the kinase [21,22,24,45]. We used IP-kinase assays and Western blotting to determine if TNF-induced proteolytic activation of PKCδ leads to sustained activation of the kinase in N27 dopaminergic cells. As shown in Figure 3A, treatment with TNF for 6 h significantly increased proteolytic cleavage of PKCδ, which was blocked by pretreatment with Etanercept or a TNFR1 receptor-neutralizing antibody. PKCδ IP-kinase assays performed in the absence of lipid cofactors showed a robust increase in PKCδ kinase activity in TNF-treated samples, which could be attenuated by pretreatment with etanercept or by TNFR1 receptor neutralization (Figure 3C). These results demonstrate for the first time that PKCδ can be proteolytically activated in dopaminergic cells by a TNFR1-dependent signaling pathway, resulting in sustained activation of the kinase during TNF-induced cell death.

### PKCδ translocates to the nucleus during TNF-induced cell death

**Figure 4:**
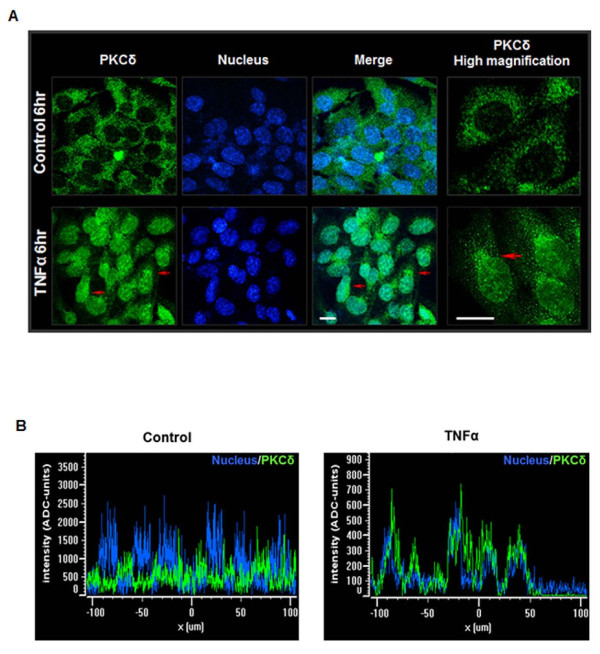
**Protein kinase Cδ (PKCδ) translocates to the nucleus during tumor necrosis factor (TNF) treatment. (A)** Confocal imaging of PKCδ localization. N27 cells were treated with TNF for 6 h and processed for immunocytochemistry using a rabbit polyclonal antibody that recognizes both the native and cleaved form of PKCδ. An Alexa-488 dye-tagged secondary antibody was used to visualize PKCδ (green), and the nucleus was stained using the TOPRO-3 dye (blue). In untreated controls (top panel), PKCδ was distinctly localized to the cytosol. In TNF-treated cells (lower panel), a distinct localization of PKCδ to the nucleus and the perinuclear region is seen. Scale bars are 20 microns. High magnification images (right panel) of PKCδ immunofluorescence staining in N27 cells showing PKCδ accumulation within the nucleus and at distinct spatial locations at the perinuclear region following TNF treatment (red arrows). Representative images are shown; experiments were repeated three times using different secondary antibodies for validation. **(B)** Fluorescence spatial intensity plots along a representative XY plane for PKCδ localization (green channel) and the nucleus (blue channel) were obtained using the Nikon EZC1 image analysis software. Control images show a distinct spatial localization of green and blue fluorescence intensities in the XY plane indicative of cytosolic PKCδ localization. In TNF-treated cells a spatial colocalization of green and blue fluorescence signals is evident indicating nuclear translocation of PKCδ.

Nuclear translocation of PKCδ has been shown to occur following proteolytic activation by apoptotic stimuli. A bipartite nuclear localization sequence (NLS) has been identified at the C-terminus (amino acids 611 to 623) of PKCδ [46,47]. The NLS is required for nuclear localization of both the full-length and proteolytically-cleaved forms of PKCδ. However, previous studies have shown that proteolytic activation of PKCδ by caspase-3 facilitates unmasking of the NLS, resulting in increased nuclear translocation of the catalytic fragment during apoptosis [47-49]. To further characterize the role of PKCδ in dopaminergic cell death induced by TNF, we used confocal microscopy to study the subcellular localization of PKCδ. N27 cells were treated with TNF for 6 h, the time point at which the highest level of cleaved PKCδ was detected, and confocal immunofluorescence was used to visualize the subcellular localization of PKCδ. We detected prominent nuclear localization of PKCδ after 6 h in TNF-treated cells, while in control cells PKCδ localized in the cytoplasm (Figure 4A). Nuclear translocation was also clearly evident in spatial fluorescence signal intensity plots (Figure 4B) for PKCδ (green channel) and the Hoechst nuclear stain (blue channel). Control cells had spatially distinct fluorescence signals for the nucleus and PKCδ, showing that PKCδ is largely localized outside the nucleus. However in TNF-treated cells, the spatial fluorescence signals had the same pattern along the XY plane. Interestingly, in addition to the overall increase in nuclear localization with TNF treatment, we also observed punctuate staining of PKCδ at distinct locations around the perinuclear region (shown in 60 × magnification and labeled with red arrows). The nuclear localization of PKCδ following TNF treatment further points to an important role for this kinase in dopaminergic degeneration during TNF toxicity.

### Overexpression of a cleavage-resistant mutant (PKCδ^D327A^-CRM) or siRNA knockdown of PKCδ protects against TNF-induced dopaminergic cell death

**Figure 5:**
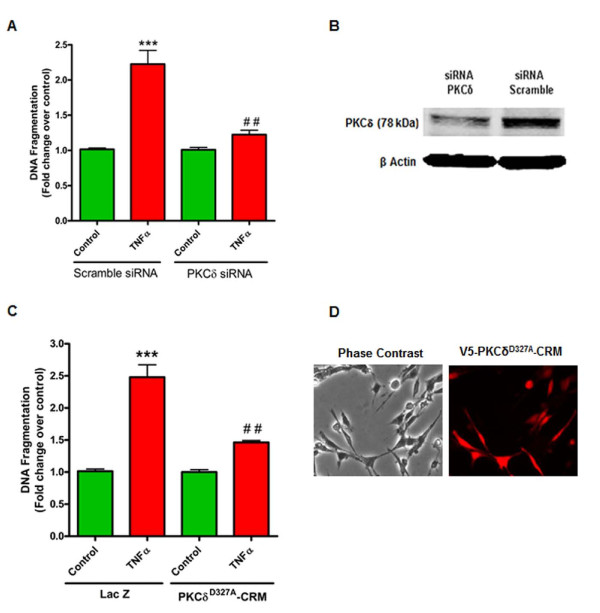
**Knockdown of protein kinase Cδ (PKCδ) with siRNA and overexpression of the PKC**δ **proteolytic cleavage-resistant mutant (PKCδ**^**D327A**^**-CRM) protects against tumor necrosis factor (TNF) toxicity. (A)** DNA fragmentation assay in siRNA transfected cells. N27 cells transfected with either siRNA to PKCδ or scrambled control siRNA were treated with TNF for 16 h and processed for the DNA fragmentation ELISA. Raw values were normalized to protein concentrations and expressed as the fold change over the respective controls. TNF-induced DNA fragmentation was significantly reduced in PKCδ siRNA transfected cells. **(B)** Suppression of the PKCδ protein levels by siRNA was confirmed by Western blotting. **(C)** DNA fragmentation assay in N27 cells overexpressing the cleavage-resistant PKCδ mutant protein (PKCδ^D327A^-CRM). N27 cells stably expressing the cleavage-resistant PKCδ mutant or the β-galactosidase (LacZ) control gene were treated with TNF for 16 h and processed for the DNA fragmentation assay. Expression of the mutant PKCδ protein was confirmed by imaging the V5 tag by immunocytochemistry. **(D)** DNA fragmentation induced by TNF was attenuated in N27 cells expressing the caspase-3 cleavage-resistant mutant protein, indicating the proteolytic cleavage is a necessary event for TNF toxicity in these cells. Data represent the group mean ± SEM, n = 6 to 8 per group and experiments were repeated three times. *** (*P* <0.001) indicates significant differences with TNF treatment compared to controls in scramble siRNA and LacZ expressing cells; ## (*P* <0.01) indicates significant differences between the two TNF treatment groups.

To determine whether TNF-induced PKCδ activation contributes to the cell death process in dopaminergic cells, we used siRNA suppression of PKCδ and a caspase-3 cleavage-resistant mutant of PKCδ (PKCδ^D327A^-CRM). First, N27 cells were transfected with siRNA to PKCδ or scramble siRNA and incubated for 24 h to allow for optimal suppression of PKCδ protein levels, which we validated by Western blotting (Figure 5B). The transfected cells were then treated with TNF for 16 h and processed for the DNA fragmentation assay. In N27 cells transfected with scramble siRNA, TNF caused a significant increase in DNA fragmentation, while the PKCδ siRNA transfected cells were protected from cell death (Figure 5A). These results indicate that PKCδ is required for TNF-induced apoptosis in dopaminergic cells and that genetic or pharmacological modulation of PKCδ may effectively attenuate TNF-induced neurotoxicity. To evaluate the significance of the PKCδ proteolytic cleavage events during proapoptotic TNF signaling, we used N27 cells overexpressing a mutant PKCδ protein resistant to proteolytic cleavage (PKCδ^D327A^-CRM) by caspase-3 due to a point mutation at the cleavage site. N27 cells that stably express either the cleavage-resistant PKCδ mutant or the LacZ protein (Figure 5D) were established as described previously [32]. The cells were treated with TNF for 16 h and processed for DNA fragmentation. As shown in Figure 5C, TNF treatment caused a 2.5-fold increase in DNA fragmentation in LacZ cells, while cells expressing the PKCδ^D327A^-CRM mutant were protected from TNF toxicity, indicating that proteolytic cleavage of PKCδ is crucial for TNF-induced cell death. Together, these results demonstrate that PKCδ functions as a proapoptotic kinase during TNF-induced dopaminergic neurotoxicity and that proteolytic cleavage of PKCδ by caspase-3 downstream of TNF signaling is required for cell death.

### Primary dopaminergic neurons obtained from PKCδ knockout (−/−) mice are protected from TNF neurotoxicity

**Figure 6:**
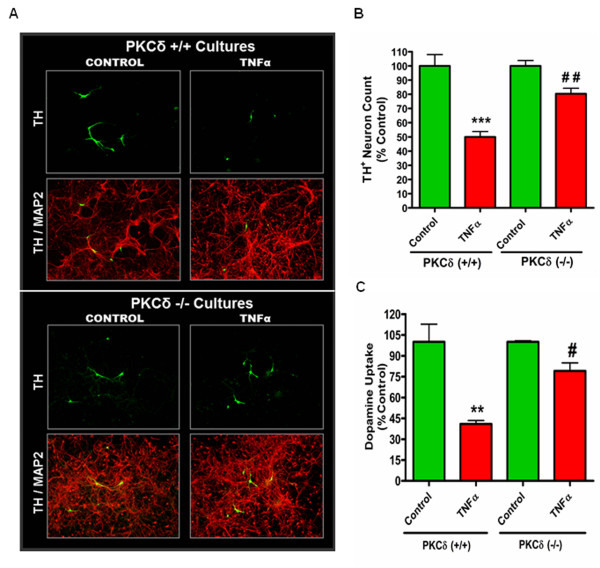
**Primary dopaminergic neurons in EVM cultures from protein kinase Cδ knockout (PKCδ −/−) mice are protected against tumor necrosis factor (TNF) toxicity.** Primary mouse EVM neuronal cultures were treated with recombinant TNF (30 ng/ml) in supplement-free neurobasal medium. TNF was re-added 24 h later for a total treatment time of 48 h. PKCδ wild-type (PKCδ +/+) and knockout (PKCδ −/−) EVM cultures were treated in parallel. **(A)** Immunocytochemistry for tyrosine hydroxylase (TH)-positive neurons. Dopaminergic neurons were identified by TH staining (green) and microtubule-associated protein 2 (MAP-2) (red) was used as a pan-neuronal marker. Images were acquired at 20 × magnification. Representative fields are shown for each treatment. In the TNF-treated group, extensive loss of neurites and degeneration of dopaminergic neurons is evident in cultures from PKCδ wild-type (+/+) mice (top panel) whereas cultures from PKCδ knockout (−/−) mice had visible branched neurites, more numerous cell bodies and were resistant to degeneration induced by TNF (lower panel). (B,C) TH-positive neuron counts and dopamine uptake assays. **(B)** Dopaminergic neurons were identified by tyrosine hydroxylase labeling, and the number of TH-positive neurons from 12 random fields per well were counted at 20 × magnification for each treatment group. **(C)** The uptake of tritiated [^3^ H] dopamine was determined in cultures from PKCδ wild-type (WT) and knockout mice. The values for non-specific uptake of dopamine obtained in the presence of mazindol were subtracted as background and the data expressed as percent control. Data represent the group mean ± SEM, n = 4 to 6 per group and experiments were repeated three times. ***P <0.001 indicates a significant difference between control and TNF-treated groups in PKCδ wild type cultures. # (*P* <0.05) and ## (*P* <0.01) indicate a significant difference between TNF-treated groups in PKCδ wild-type and PKCδ knockout (−/−) cultures.

In order to extend our results from the previous mechanistic studies with the dopaminergic N27 clonal cell model to dopaminergic neurons, we used primary mouse ventral mesencephalic dopaminergic neuron cultures. We established primary mesencephalic neuronal (>95 % glial-free) cultures from PKCδ wild type (+/+) and knockout (−/−) mice to study the role of PKCδ in mediating TNF-induced dopaminergic neurodegeneration. Dopaminergic neurotoxicity was assessed by dopamine uptake assays and by TH-positive cell counting. As shown (Figure 6B,C), TNF exposure caused around 50 % decrease in dopamine uptake and TH-positive cell counts in cultures from PKCδ wild type (+/+) mice, consistent with previous reports of dopaminergic neurotoxicity in similar model systems [17,26]. However, in primary neuron cultures obtained from PKCδ knockout (−/−) mice, TNF induced only around a 20 % reduction in dopamine uptake and TH-positive neuron counts, indicating that dopaminergic neurons from PKCδ knockout mice were resistant to TNF toxicity. In addition to functional measurement of TNF neurotoxicity by dopamine uptake, we also performed double labeling immunofluorescence morphometric analysis using TH as a marker for dopaminergic neurons and MAP-2 as a pan-neuronal marker. Untreated cultures from both PKCδ wild type (+/+) and knockout (−/−) mice had similar numbers of dopaminergic neurons and displayed extensive branched neurites and normal cell bodies (Figure 6A). In TNF-treated cultures, fewer TH-positive neurons with smaller cell bodies and a clear loss of neurites were evident in cultures from PKCδ wild type (+/+) mice. In contrast, dopaminergic neurons in cultures from PKCδ knockout (−/−) mice displayed visible branched neurites and more numerous cell bodies with normal morphology (Figure 6A lower panel), demonstrating significant protection against TNF toxicity in these cultures.

### PKCδ is activated by proteolytic cleavage in the mouse substantia nigra following stereotaxic infusion of LPS

**Figure 7:**
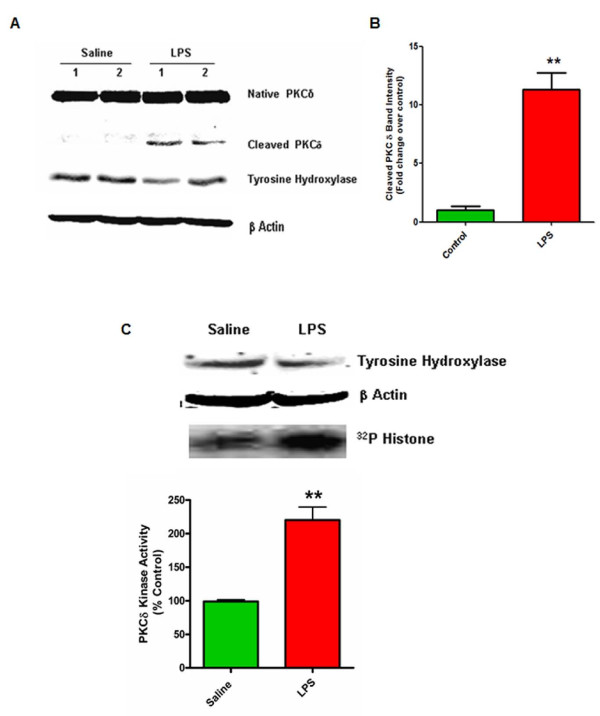
**Proteolytic activation of protein kinase Cδ (PKCδ) in the mouse substantia nigra during LPS-induced dopaminergic degeneration.** C57BL/6 mice were stereotaxically injected with a single dose of lipopolysaccharide (LPS) (5 μg) or saline in the substantia nigra (SN) to elicit dopaminergic degeneration. Mice were sacrificed after 14 days. **(A)** Proteolytic cleavage of PKCδ in mouse substantia nigra tissue. Western blots were performed on nigral tissue lysates obtained from mice stereotaxically injected with either LPS or saline. Strong proteolytic cleavage of PKCδ is seen in LPS-injected mice and was essentially absent in the saline-injected animals. Blots were also probed for tyrosine hydroxylase and β-actin to confirm accurate dissection of the nigral tissue and equal protein loading respectively. **(B)** PKCδ cleaved band intensity was quantified by densitometric analysis after normalization to β-actin (n = 4 to 6 mice per group). **(C)** PKCδ kinase activity in mouse substantia nigra tissue. PKCδ IP-kinase assays were performed on nigral tissue lysates. Kinase activity was quantified by densitometric analysis of the ^32^P-histone bands (n = 4 mice per group). A representative kinase gel is shown. Western blots of tyrosine hydroxylase and β-actin were performed on the same lysates to verify accurate dissection of the nigral tissue. Data represent the group mean ± SEM. ** (*P* <0.01) indicates a significant difference between saline and LPS-injected mice.

Next, we sought to extend the significance of our results obtained with the N27 dopaminergic cell culture model and primary neurons to an in vivo model of dopaminergic degeneration relevant to neuroinflammation. We selected the widely used stereotaxic LPS injection model of neuroinflammation-induced dopaminergic degeneration for our experiments [17,35,50,51]. In this model, a single injection of LPS into the rodent substantia nigra elicits a sustained, localized neuroinflammatory response resulting in a delayed loss of dopaminergic neurons in the SNpc. We stereotaxically injected C57bl6 mice with a single dose of 5 μg LPS into the substantia nigra and sacrificed them 14 days later, the time at which significant dopaminergic degeneration occurs in this model [35]. The SN tissue was dissected and probed for proteolytic activation of PKCδ using Western blotting and IP-kinase assays. As shown in Figure 7A, mice that received saline injections did not show proteolytic activation of PKCδ in the SN, while LPS-injected mice showed strong proteolytic cleavage of PKCδ at 14 days. The PKCδ Western blots were reprobed for tyrosine hydroxylase to confirm dissection of the substantia nigra region. As expected, the LPS-injected mice had decreased TH protein levels (Figure 7A), indicative of increased dopaminergic degeneration in this model at 14 days [35,50]. We also used PKCδ IP-kinase assays with a histone substrate on nigral tissue samples from LPS-injected and saline-injected mice. In nigral tissues from LPS-injected mice, we detected substantially higher PKCδ kinase activity compared to saline injected controls (Figure 7C), demonstrating that the proteolytic cleavage of PKCδ resulted in activation of the kinase in vivo. Equal protein loading and accurate dissection of the nigral tissue was confirmed by Western blotting for β-actin and TH, respectively. Together, these results demonstrate for the first time that protein kinase Cδ can be activated by proteolytic cleavage in the substantia nigra following LPS-induced neuroinflammation.

## Discussion

**Figure 8:**
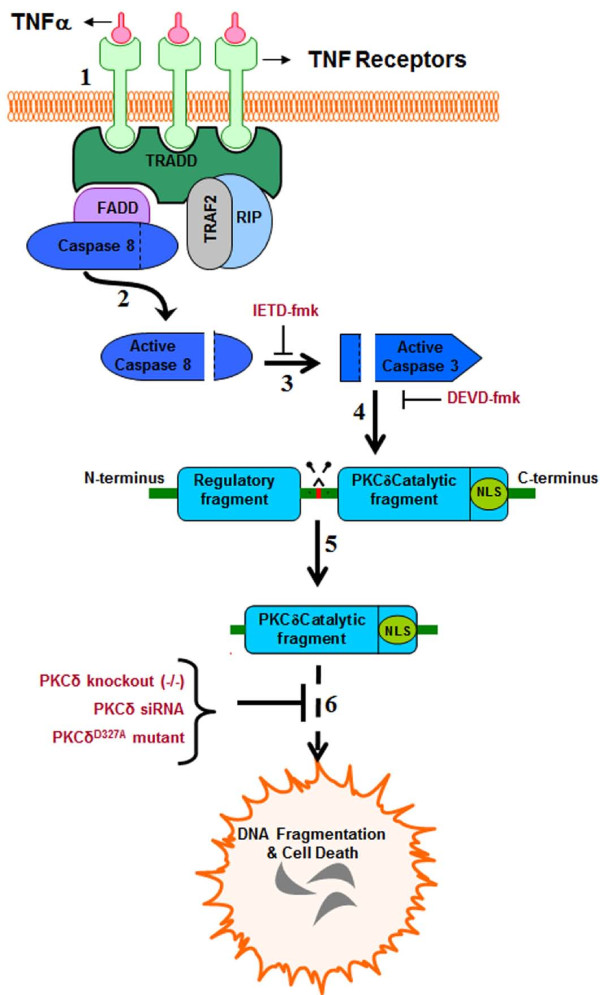
**Schematic of protein kinase Cδ (PKCδ) activation by proteolytic cleavage downstream of tumor necrosis factor (TNF) signaling in dopaminergic neurons. (1)** Sustained TNF signaling leads to caspase-8 activation, presumably by autoproteolytic cleavage at the receptor complex **(2)**. Active caspase-8 leads to downstream activation of caspase-3, which can be blocked by the caspase-8 inhibitor IETD-fmk **(3)**. Caspase-3 activates PKCδ by proteolytic cleavage **(4)** at the hinge region (shown in red), releasing the catalytic fragment and causing constitutive activation of the kinase **(5)**. The constitutively active PKCδ catalytic fragment mediates proapoptotic signaling by phosphorylation of its downstream substrates, resulting in DNA fragmentation and dopaminergic cell death **(6)**. Blocking the PKCδ signaling pathway using siRNA, targeted gene knockout or a caspase-3 cleavage-resistant mutant can protect against dopaminergic cell death induced by TNF toxicity.

In this study, we identify PKCδ as a novel signaling mediator downstream of TNF-induced toxicity in dopaminergic neurons and demonstrate that proteolytic activation of PKCδ regulates dopaminergic degeneration induced by TNF (Depicted schematically in Figure 8). We also demonstrate for the first time, the proteolytic activation of PKCδ in the mouse substantia nigra by neuroinflammatory mechanisms during LPS-induced dopaminergic degeneration. The proteolytic activation of PKCδ by neuroinflammatory insults via the extrinsic apoptotic pathway has not been studied, therefore it is essential to elucidate the mechanisms underlying progressive degeneration of dopaminergic neurons by chronic neuroinflammatory processes involving TNF and other neurotoxic mediators [52,53]. Based on the substantial literature supporting a degenerative role for TNF during dopaminergic cell death in PD models, we hypothesized that PKCδ can be proteolytically activated downstream of canonical TNF death receptor signaling by caspase-3 in dopaminergic neurons. Since proteolytic cleavage of PKCδ by caspase-3 typically results in persistent activation of the kinase as an apoptotic effector, we reasoned that blocking proapoptotic PKCδ signaling could protect against TNF toxicity.

The results from our mechanistic studies using N27 rat dopaminergic cells indeed demonstrate for the first time that soluble TNF induces a time-dependent and dose-dependent proteolytic cleavage of PKCδ by a caspase-8 and caspase-3 signaling pathway, indicative of canonical TNF death receptor signaling via caspase-8. Using the anti-TNF drug Etanercept and neutralizing antibodies to the TNFR1 (p55) death receptor, we show that proteolytic cleavage of PKCδ by soluble TNF is dependent on TNFR1 signaling and is accompanied by a substantial increase in PKCδ kinase activity (Figure 3), consistent with proteolytic activation. Previous reports from our laboratory and other independent research groups have shown that PKCδ proteolytic activation contributes to apoptosis induced by diverse neurotoxic stimuli that converge on the intrinsic mitochondrial apoptotic pathway primarily through intracellular oxidative stress mechanisms [21-24,31,32,54-56]. Taken together, our present results suggest that proteolytic activation of PKCδ could be a common effector of cell death via both intrinsic and extrinsic cascades in dopaminergic neurons.

Rapid and dynamic translocation to distinct subcellular locations following activation is a characteristic feature of PKCδ in different cell types, and typically delineates the unique functional role of this kinase [57]. Various substrates have been identified as targets of PKCδ phosphorylation in different subcellular locations that participate in a range of multiple cellular functions, from apoptosis to cell migration and activation of immune cells [58]. In particular, studies have demonstrated that nuclear translocation of PKCδ is required for induction of apoptosis and is driven by a bipartite nuclear localization sequence at its C-terminus [48,49,59]. Our confocal immunofluorescence experiments reveal a distinct localization of PKCδ to the nucleus 6 h after TNF treatment, the time point at which maximum proteolytic cleavage of PKCδ was evident, consistent with its proapoptotic function (Figure 4). While intense PKCδ staining is evident throughout the nucleus in TNF-treated cells, there is also a distinct pattern of accumulation of PKCδ at specific sites around the perinuclear region and within the nucleus. Interestingly, in a previous study, TNF superfamily ligands, such as BAFF (B cell-activating factor belonging to the TNF family), have been shown to prevent nuclear accumulation of full length PKCδ and thus prolong cell survival in B cells [60]. The substrates of PKCδ and the functional significance of nuclear localization of the kinase during neuroinflammation are currently under investigation in our laboratory. However, the kinetics of PKCδ proteolytic activation and its translocation to the nucleus observed in our present study, both indicate of a proapoptotic role for PKCδ downstream of TNF. Indeed, suppressing total PKCδ protein levels using siRNA or blocking proteolytic activation using a PKCδ cleavage-resistant mutant (PKCδ^D327A^-CRM) affords robust protection against TNF neurotoxicity (Figure 5). The results from our mechanistic studies are further supported by our experiments in primary mesencephalic neuronal cultures treated with soluble TNF, which induces degeneration of dopaminergic neurons in these cultures [17,26]. Although TNF was less toxic to primary dopaminergic neurons than the classical Parkinsonian neurotoxicant 1-methyl-4-phenylpyridinium (MPP^+^) [21], we observed a substantial reduction in TH neuron numbers and functional impairment as determined by dopamine uptake studies in cultures from PKCδ wild type (+/+) mice (Figure 6). Importantly, dopaminergic neurons in mesencephalic cultures obtained from PKCδ knockout (−/−) mice were protected against TNF toxicity, indicating that PKCδ signaling is essential for dopaminergic degeneration triggered by TNF.

Evidence from previous studies [17,26] and our own results here, indicate that TNF can induce dopaminergic neurotoxicity by a direct mechanism, possibly via the TNFR1 (TNFRSF1) death receptor, which has been shown to be highly expressed on these cells. However, additional lines of evidence suggest that TNF can also contribute to dopaminergic degeneration indirectly by potentiating microglial reactive oxygen species (ROS) and other neurotoxic mediators, which can increase local oxidative stress levels in the SNpc [19,52,61]. The elevated TNF levels in the SN in both animal models and human PD patients and the concomitant microglial activation indicate that potentiation of microglial neurotoxic responses by TNF likely occurs in vivo, although it may not be the primary mechanism of dopaminergic neurotoxicity, as suggested by McCoy et al. using mixed neuron-glial cultures. A chronic increase in TNF levels in the nigrostriatal system may drive the progressive loss of compromised and vulnerable dopaminergic neurons in the pro-oxidant environment of the SNpc, both by TNF apoptotic signaling and by potentiation of microglial neurotoxic responses, including ROS and RNS production [17,19,26,52,62]. Nonetheless, our data demonstrating that PKCδ is proteolytically activated in the substantia nigra in the neuroinflammatory LPS model (Figure 7) of PD, implicate proapoptotic PKCδ signaling as a common downstream effector of dopaminergic cell death triggered by convergent neuroinflammatory mechanisms involving TNF and other neurotoxic mediators in the mouse brain.

## Conclusions

Despite extensive research into the etiology of PD for several decades, a cogent basis for the vulnerability and extensive loss of SNpc dopaminergic neurons still remains elusive. The emerging consensus is that PD results from complex interactions between environmental, genetic and cellular processes that induce dopaminergic cell death over time [3,63]. This highlights the multifactorial nature of the disease process and underscores the importance of identifying therapeutic targets that are relevant across these multiple mechanisms of disease pathogenesis. While oxidative stress, mitochondrial dysfunction and proteasomal impairment have been identified as causative factors that can trigger dopaminergic cell death; reactive microgliosis and dysregulated neuroinflammation can sustain this process and result in progressive neurodegeneration [6,52,53]. We previously showed that proteasomal impairment, mitochondrial dysfunction and oxidative stress induce proteolytic activation of PKCδ to drive dopaminergic cell death [24,45,64]. Our results herein suggest a crucial role for proapoptotic PKCδ signaling during dopaminergic cell death induced by extracellular TNF and neuroinflammatory mechanisms. Pharmacological targeting of PKCδ with kinase inhibitors such as rottlerin has shown efficacy in protecting against dopaminergic degeneration in vitro and in the MPTP mouse model of PD [21,55]. Our results from this study provide a rationale for pharmacological targeting of PKCδ to mitigate neuroinflammatory events associated with progressive degeneration of dopaminergic neurons in PD. Taken together, our results herein suggest that proteolytic activation of PKCδ could be a common downstream target for multiple mediators of dopaminergic degeneration by both extrinsic and intrinsic cell death mechanisms, making it an attractive therapeutic target during the progressive phase of PD.

## Competing interests

The authors declare that they have no competing interests.

## Authors’ contributions

RG designed studies, performed experiments and wrote the manuscript. VA assisted with study design and manuscript editing. AGK and AK conceived the study, the coordination of experiments and editing of the manuscript drafts. All authors were involved in editing drafts of the manuscript and approved the final manuscript.
